# APEX3 – an optimized tool for rapid and unbiased proximity labeling

**DOI:** 10.1016/j.jmb.2023.168145

**Published:** 2023-05-13

**Authors:** Jordan T. Becker, Ashley A. Auerbach, Reuben S. Harris

**Affiliations:** 1 Department of Biochemistry, Molecular Biology, and Biophysics, University of Minnesota Twin Cities, Minneapolis, MN, USA 55455; 2 Department of Microbiology and Immunology, University of Minnesota Twin Cities, Minneapolis, MN, USA 55455; 3 Institute for Molecular Virology, University of Minnesota Twin Cities, Minneapolis, MN, USA 55455; 4 Department of Biochemistry and Structural Biology, University of Texas Health San Antonio, San Antonio, TX, USA 78229; 5 Howard Hughes Medical Institute, University of Texas Health San Antonio, San Antonio, TX, USA 78229

**Keywords:** APEX2/3, nuclear export signal (NES), nucleocytoplasmic shuttling, proximity labeling technology, subcellular localization

## Abstract

Macromolecular interactions regulate all aspects of biology. The identification of interacting partners and complexes is important for understanding cellular processes, host-pathogen conflicts, and organismal development. Multiple methods exist to label and enrich interacting proteins in living cells. Notably, the soybean ascorbate peroxidase, APEX2, rapidly biotinylates adjacent biomolecules in the presence of biotin-phenol and hydrogen peroxide. However, during initial experiments with this system, we found that APEX2 exhibits a cytoplasmic-biased localization and is sensitive to the nuclear export inhibitor leptomycin B (LMB). This led us to identify a putative nuclear export signal (NES) at the carboxy-terminus of APEX2 (NES_APEX2_), structurally adjacent to the conserved heme binding site. This putative NES is functional as evidenced by cytoplasmic localization and LMB sensitivity of a mCherry-NES_APEX2_ chimeric construct. Single amino acid substitutions of multiple hydrophobic residues within NES_APEX2_ eliminate cytoplasm-biased localization of both mCherry-NES_APEX2_ as well as full-length APEX2. However, all but one of these NES substitutions also compromises peroxide-dependent labeling. This unique separation-of-function mutant, APEX2-L242A, is termed APEX3. Localization and functionality of APEX3 are confirmed by fusion to the nucleocytoplasmic shuttling transcriptional factor, RELA. APEX3 is therefore an optimized tool for unbiased proximity labeling of cellular proteins and interacting factors.

## Graphical Abstract

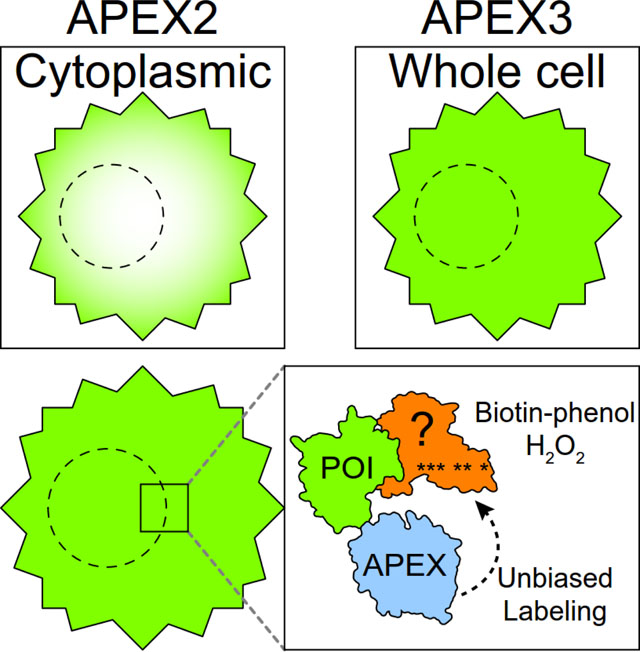

## INTRODUCTION

The subcellular localization of biological macromolecules is a key determinant of function [1–6]. Notably, RNA-binding proteins (RBPs) and their preferred RNA substrates must occupy or transit to the same subcellular location in order to interact [7–10]. In homeostatic circumstances, colocalization is important for regulating key biological processes including RNA trafficking in neurons, mRNA translation, splicing, RNA export, and non-sense mediated decay [11–15]. In the context of host-pathogen conflicts, compartmentalized interactions are required for multiple antiviral mechanisms such as APOBEC3 packaging into retroviral virions [16,17] and ZAP binding to CpG-rich RNAs [18–20]. Finally, these interactions determine successful or abortive virus replication (*e.g*., viral structural proteins binding genomic RNA or RNA polymerase with template viral RNA [21,22]). Furthermore, purposeful disruption of biological localization is a pharmaceutical target (*e.g*., the XPO1 inhibitor Selinexor disrupts nuclear export) [23–27]. Understanding the diverse interactions of a particular protein of interest (POI) is important and, accordingly, multiple methods have been developed to interrogate these interactions.

Protein-protein interactions (PPIs) and RNA-protein interactions (RPIs) are commonly investigated by immunoprecipitation (IP) of a known target using a specific antibody coupled to downstream mass spectrometry or RNA sequencing, respectively [28]. These methods are effective but may sometimes miss relevant interactions. For example, a particular protein may only interact with some partners transiently, weakly, or indirectly (*i.e*., through interaction with other molecules). Recent advances for *in cellulo* proximity labeling allow for tunable labeling and enrichment of interacting molecular complexes as well as capturing of transient interactions. Notably, the engineered soybean ascorbate peroxidase (APEX2) catalyzes the conjugation of biotin-phenol to adjacent proteins and RNA in the presence of hydrogen peroxide [29–31]. This reaction occurs rapidly (seconds to minutes) and allows for flexible experimental modification. Indeed, APEX2 has been used to identify the spatiotemporal PPI network of GPCR signaling [32], the spatiotemporal PPI and RPI networks of RNA granule formation [33], and a transcriptome-wide atlas of subcellular RNA localization [34]. Notably, these studies used proteins with strong subcellular localization determinants fused to APEX2.

We initially intended to use APEX2 to identify the PPI and RPI networks and shuttling mechanisms of a family of related cellular proteins with disparate nuclear and cytoplasmic localizations. However, during experiments designed to validate the localization of APEX2 fusion proteins we found that APEX2 itself exhibits a cytoplasm-biased localization rather than a diffuse, whole-cell localization that is characteristic of most fluorescent and non-compartmentalized proteins. This observation led to the identification of a putative nuclear export signal (NES) in APEX2 that is conserved in the parental soybean ascorbate peroxidase 1 (APX1) protein as well other plant homologs. The functionality of this putative NES is demonstrated by cytoplasmic localization of a heterologous fusion to mCherry and responsiveness to the XPO1 inhibitor leptomycin B (LMB). In addition, mutations to hydrophobic residues in this NES motif compromise the cytoplasm-biased localization of APEX2, however most of these mutants also lack peroxidase activity. We identified one APEX2 mutant (L242A) that exhibits whole-cell localization and retains peroxidase activity. This separation-of-function mutant, termed APEX3, is further validated by fusion to a well-characterized nucleocytoplasmic shuttling protein, the RELA component of NF-κB. Overall, we describe an optimized proximity labeling technology, APEX3, that expands the utility of this technology and may help answer a broader number of important questions.

## RESULTS

### APEX2 exhibits a cytoplasm-biased localization

**Figure 1.**
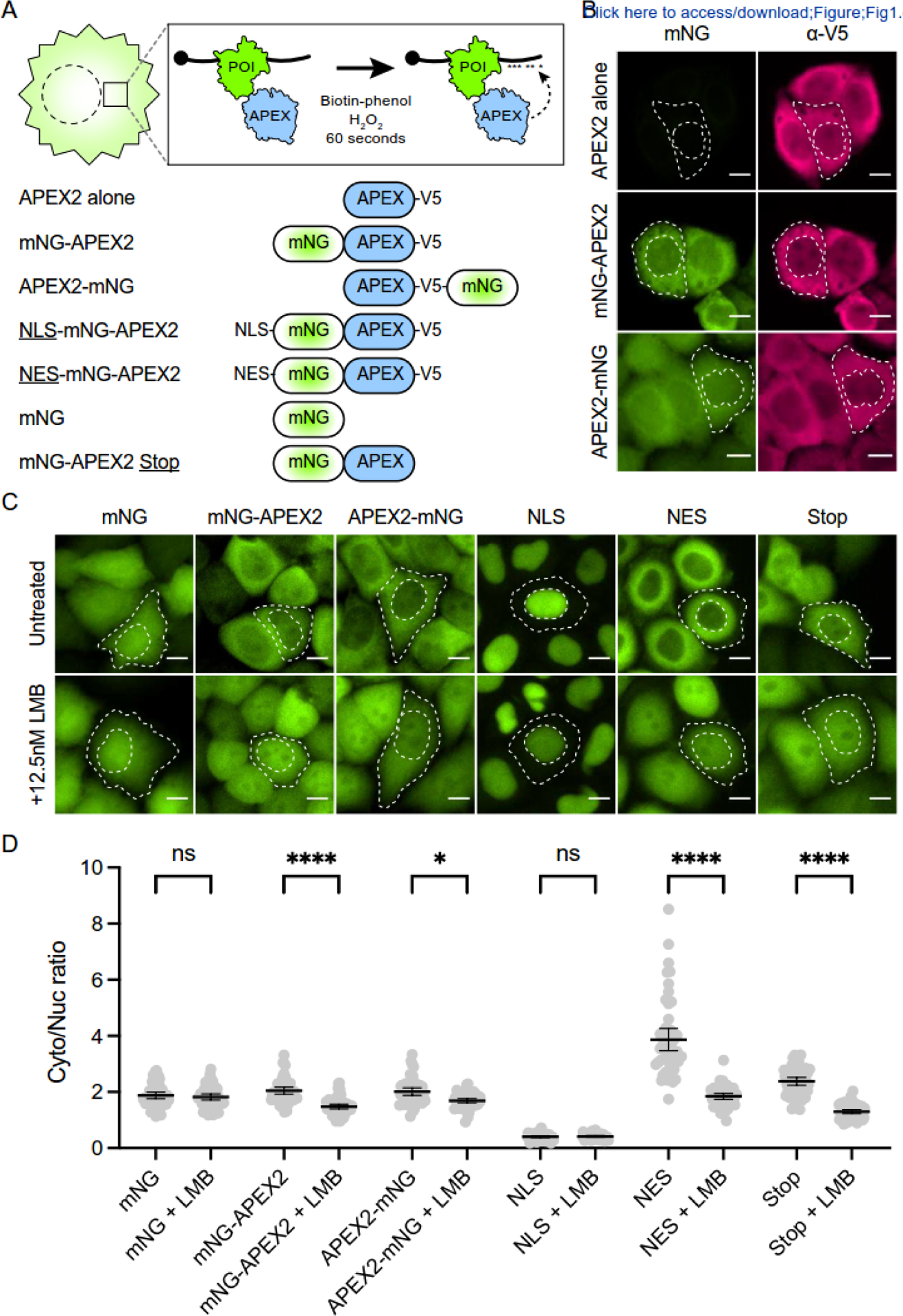
APEX2 is cytoplasmic and leptomycin B sensitive. (**A**) Schematic of APEX2-dependent biotin labeling of a protein-of-interest (POI). Illustrations of constructs used in Figure 1 and Figure S1 studies. (**B**) Representative fixed images of the indicated APEX2 constructs expressed stably in HeLa cells (green, mNeonGreen fluorescence; rose, anti-V5 immunostaining; scale = 10 μm). Cell and nuclear boundaries outlined with white dashed lines. (**C**) Representative live-cell images of the indicated APEX2 constructs expressed stably in HeLa cells following mock (top) or 2 hrs LMB treatment (bottom) (green, mNeonGreen fluorescence; scale = 10 μm). Cell and nuclear boundaries outlined with white dashed lines. (**D**) Quantification of cytoplasmic-to-nuclear mNeonGreen fluorescence ratio in experiments from panel C (N=50 cells per condition; ns, not significant; *, *p* < 0.05; ****, *p* < 0.0001 by one-way ANOVA; scale = 10 μm).

We were initially interested in using APEX2 to identify interacting factors for nucleocytoplasmic shuttling proteins. In the early stages of these experiments, we generated control constructs for labeling different subcellular compartments (*i.e*., nucleus, cytoplasm, and whole cell; construct schematics in Figure 1A): APEX2 alone, APEX2 fused to mNeonGreen (mNG) at either its amino- or carboxy-terminus (mNG-APEX2 or APEX2-mNG), a strong NLS [35] fused to mNG-APEX2 (NLS-mNG-APEX2), and a strong NES [36–38] fused to mNG-APEX2 (NES-mNG-APEX2). A V5 epitope tag was also added to the carboxy-terminus of APEX2 in all these constructs for detection. HeLa cell lines were generated by transduction to stably express each of these constructs for imaging by fluorescence microscopy. To our surprise, we noticed that APEX2 alone as well as mNG-APEX2 and APEX2-mNG exhibit greater cytoplasmic than nuclear fluorescence intensity (Figure 1B). This localization bias was evident in fixed cells using direct mNG fluorescent signal (green) as well as by indirect V5 epitope staining and immunofluorescent microscopy (rose).

As expected, the NES-mNG-APEX2 control construct with a heterologous NES exhibits increased nuclear localization in HeLa cells following leptomycin B (LMB) treatment to inhibit XPO1-dependent nuclear export [39]. To our surprise, the mNG-APEX2 and APEX2-mNG constructs (without a heterologous NES) also exhibit increased nuclear localization following LMB treatment (Figure 1C, quantified in 1D). As additional controls, mNG alone and NLS-mNG-APEX2 are unaffected by LMB. To demonstrate that this apparent LMB-sensitive (XPO1-dependent) nuclear export activity is intrinsic to APEX2 and not to other features of the mNG-APEX2-V5 fusion constructs, we created a mNG-APEX2 “Stop” construct lacking the V5 epitope and found that it still exhibits cytoplasmic localization and sensitivity to LMB treatment (Figure 1C, quantified in 1D). These constructs also display similar subcellular localization properties in 293T cells (Figure S1). Thus, these results led us to hypothesize that APEX2 has a signal or motif responsible for the observed cytoplasm-biased localization.

### APEX2 encodes an amino acid motif that acts as a heterologous NES

**Figure 2.**
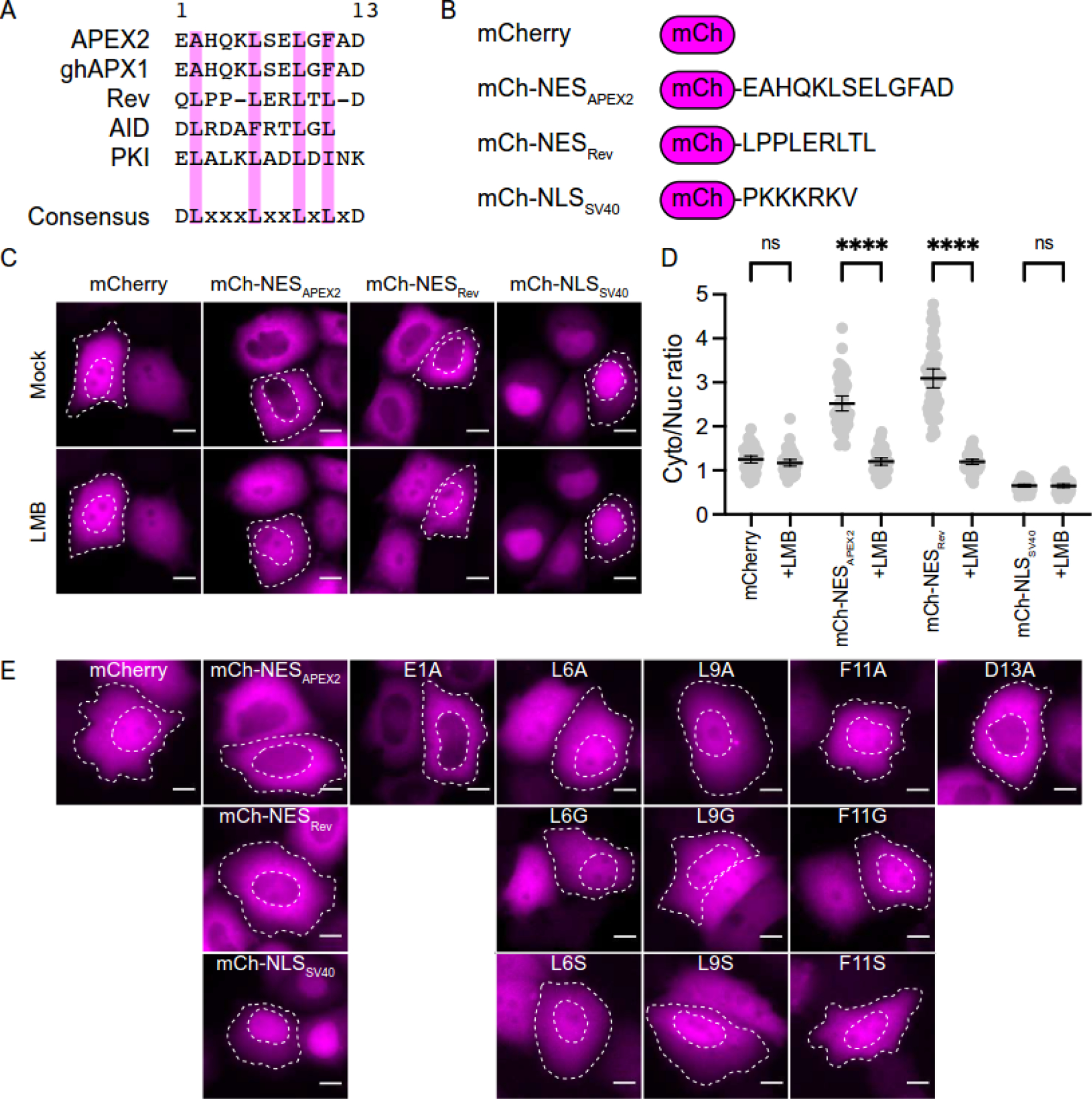
Putative APEX2 NES functions autonomously upon fusion to mCherry. (**A**) Amino acid alignment of established NES peptides, NES_APEX2_, and motif found in *Glycine hispida apx1*. (**B**) Schematic of constructs used in Figure 2. (**C**) Representative live-cell images of the indicated mCherry constructs expressed transiently in HeLa cells following mock (top) or 2 hrs LMB treatment (bottom) (pink, mCherry fluorescence; scale = 10 μm). Cell and nuclear boundaries outlined with white dashed lines. (**D**) Quantification of cytoplasmic-to-nuclear mCherry fluorescence ratio in experiments from panel C (N=50 cells per condition; ns, not significant; ****, *p* < 0.0001 by one-way ANOVA; scale = 10 μm). (**E**) Representative fixed images of HeLa cells transfected with indicated mCherry or mCherry-NES constructs. The single amino acid substitution mutants (sequence in panel A) are derivatives of mCherry-NES_APEX2_ (scale = 10 μm). Cell and nuclear boundaries outlined with white dashed lines.

Many XPO1-dependent nucleocytoplasmic cargo proteins have an amino acid motif with 3–4 hydrophobic residues [40–42], which is often flanked by acidic residues [43] (*e.g*., DxLxxxLxxLxLxD; alignment in Figure 2A). Submission of the APEX2 amino acid sequence (including mNG, peptide linkers, and V5 epitope) to multiple NES prediction servers [44–46] did not identify such a motif. However, manual inspection of the primary APEX2 amino acid sequence revealed an NES-like region at the carboxy-terminus of the protein that is shared with the parental soybean (*Glycine hispida*) ascorbate peroxidase, ghAPX1 (Figure 2A). Alignment of hundreds of available plant APX1 homologs indicated that this putative NES is highly conserved with the central leucine residues demonstrating 100% conservation (Figure S2). This putative NES is also found in split APEX2 [47] as well as the original APEX [48] derived from *Pisum sativum* APX.

To test whether this candidate NES is responsible for APEX2 cytoplasmic localization, this motif (NES_APEX2_) was fused to the carboxy-terminus of mCherry and examined in HeLa cells by fluorescent microscopy. In comparison to mCherry alone, which has a cell-wide distribution, mCherry-NES_APEX2_ exhibits a distinctly cytoplasmic localization (Figure 2B–C; quantification in Figure 2D). Moreover, LMB treatment of cells causes an accumulation of this construct in the nuclear compartment and yields an overall cell wide appearance. As a positive control, an analogous mCherry fusion construct with the well-characterized NES of HIV-1 Rev behaves similarly (Figure 2B–C; quantified in 2D). In contrast, an mCherry construct fused with the NLS of SV40 shows predominantly nuclear localization and is unaffected by LMB (Figure 2B–C; quantified in 2D).

We next generated a panel of single amino acid mCherry-NES_APEX2_ mutant constructs to determine the residues responsible for nuclear export. NES-containing cargos are exported from the nucleus by XPO1, and this interaction is typically mediated by hydrophobic residues within the NES and can be further influenced by flanking acidic residues [40,43]. Therefore, these conserved residues were changed to alanine, glycine, or serine and studied by fluorescence microscopy in HeLa cells. Mutation of the acidic residues of the NES motif resulted in proteins that maintain export function as evidenced by predominantly cytoplasmic localization (E1A or D13A in Figure 2E). In contrast, changes to hydrophobic residues L6, L9, and F11 compromised export activity, evidenced by whole-cell mCherry localization (L6A/G/S, L9A/G/S, and F11A/G/S in Figure 2E). Taken together, these results demonstrate that the putative NES of APEX2 is both portable and functional and therefore a robust export motif.

### Most APEX2 NES mutants also lack peroxidase activity

**Figure 3.**
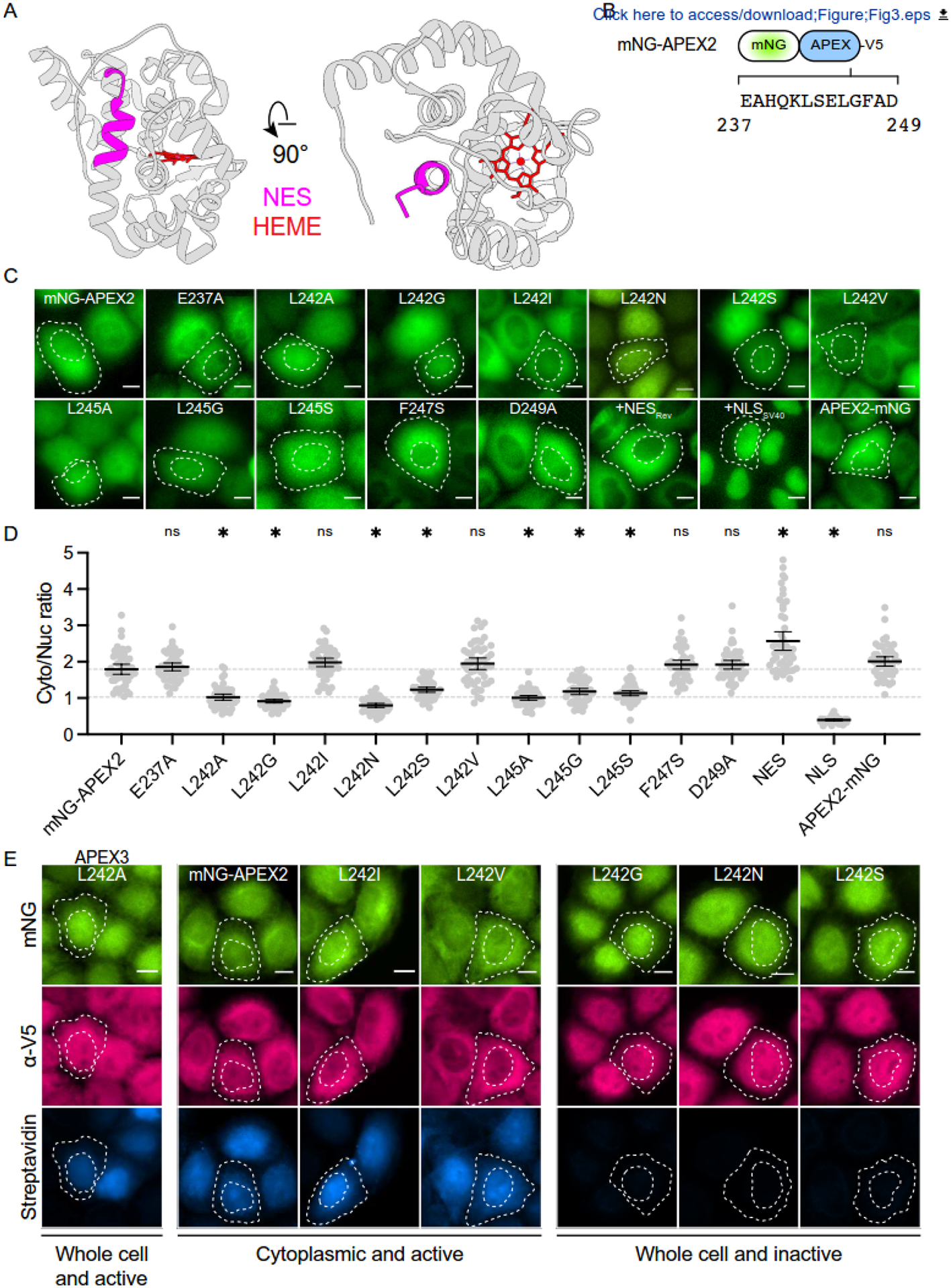
Putative APEX2 NES residues are mostly required for peroxidase activity. (**A**) Ribbon schematic of the *Glycine hispida* APX1 crystal structure (PDB: 1OAG) with NES highlighted in magenta and the heme in red. The NES in APEX2 is identical (Figure 2A). (**B**) Schematic of the mNG-APEX2-V5 construct and the approximate position of NES_APEX2_ residues. (**C**) Representative live-cell images of the indicated mNG-APEX2-V5 constructs expressed stably in HeLa cells (green, mNG fluorescence; scale = 10 μm). Cell and nuclear boundaries outlined with white dashed lines. (**D**) Quantification of cytoplasmic-to-nuclear mNeonGreen fluorescence ratio in experiments from panel C (N=50 cells per condition; ns, not significant; *, *p* < 0.0001 by one-way ANOVA compared to APEX2; scale = 10 μm). Gray dashed lines highlight values for APEX2 (top) and L242A (bottom). (**E**) Representative fixed images of mNG-APEX2-V5 and the indicated L242 mutants expressed stably in HeLa cells showing mNeonGreen fluorescence (green), anti-V5 staining (rose), and streptavidin staining to detect peroxide-dependent biotinylation (blue) (scale = 10 μm). Cell and nuclear boundaries outlined with white dashed lines.

Upon inspection of the structure of soybean ascorbate peroxidase (PDB: 1OAG) [49], we noticed that the putative NES is part of an alpha-helix typical of many NES cargos (Figure 3A). This putative NES may have eluded prediction algorithms (*e.g*., LocNES and NESsential) because it may have been deemed structurally inaccessible and unlikely to achieve the disorder/flexibility required to engage export machinery [44,46]. Indeed, the critical hydrophobic residues required for export are positioned toward the globular center of the protein and therefore are likely to contribute to structural stability and function of the overall enzyme as a peroxidase (Figure 3A). To address this possibility and ask whether the putative APEX2 NES is active in the context of its native protein structure, single amino acid substitution mutations were introduced into the NES motif of the full-length mNG-APEX2-V5 construct (Figure 3B). Most of these mutations were selected to reduce hydrophobic interactions with XPO1 [40] and to overlap with the set described above. These constructs were expressed stably in HeLa cells and compared using fluorescence microscopy.

As expected, most substitutions that eliminate NES activity in the heterologous mCherry-NES_APEX2_ construct described above also cause analogous reductions in the NES activity of full-length APEX2 (Figure 3C, quantified in 3D). Specifically, L242A/G/S/N or L245A/G/S single amino acid mutants show cell-wide localization indicative of compromised NES-like function. More conservative changes at position 242 (L242V and L242I) have no effect on NES function and, as above, the acidic residues at positions 237 and 249 are dispensable for function (E237A and D249A). An exception appears to be F247S which maintains NES function in the full-length protein despite losing it above in the heterologous mCherry-NES_APEX2_ context. Nevertheless, taken together, these results further confirm the functionality of the putative APEX2 NES.

However, when the same cell lines were subjected to hydrogen peroxide-dependent labeling with biotin-phenol, we found that only the L242A mutant of mNG-APEX2-V5 retains labeling activity as detected by streptavidin immunofluorescence (Figure 3E and Figure S3, quantified in S3C). All other mutants showed one of three different phenotypes. The first group retained wild type-like peroxide-dependent labeling activity, as well as NES-like function as evidenced by cytoplasmic localization (*e.g*., E237A, L242I/V, D249A). The second group lost both peroxide-dependent labeling activity as well as NES function (*e.g*., L242G/N/S and L245A/G/S). The third group lost peroxide-dependent labeling activity but retained NES-like function (*e.g*., F247S as well as F231A, F232A, and Y235A that are located amino-terminal relative to the putative NES motif). We speculate that this null phenotype might be due to weakened heme binding and/or a compromised core structure (despite maintaining near wildtype expression levels; Figure 3E and Figure S3). Ultimately, only one single amino acid substitution mutant, L242A, exhibited whole cell localization while retaining peroxidase-dependent labeling activity and, thus, this protein is named APEX3.

### APEX3 localizes faithfully upon fusion to nucleocytoplasmic shuttling factor RELA

**Figure 4.**
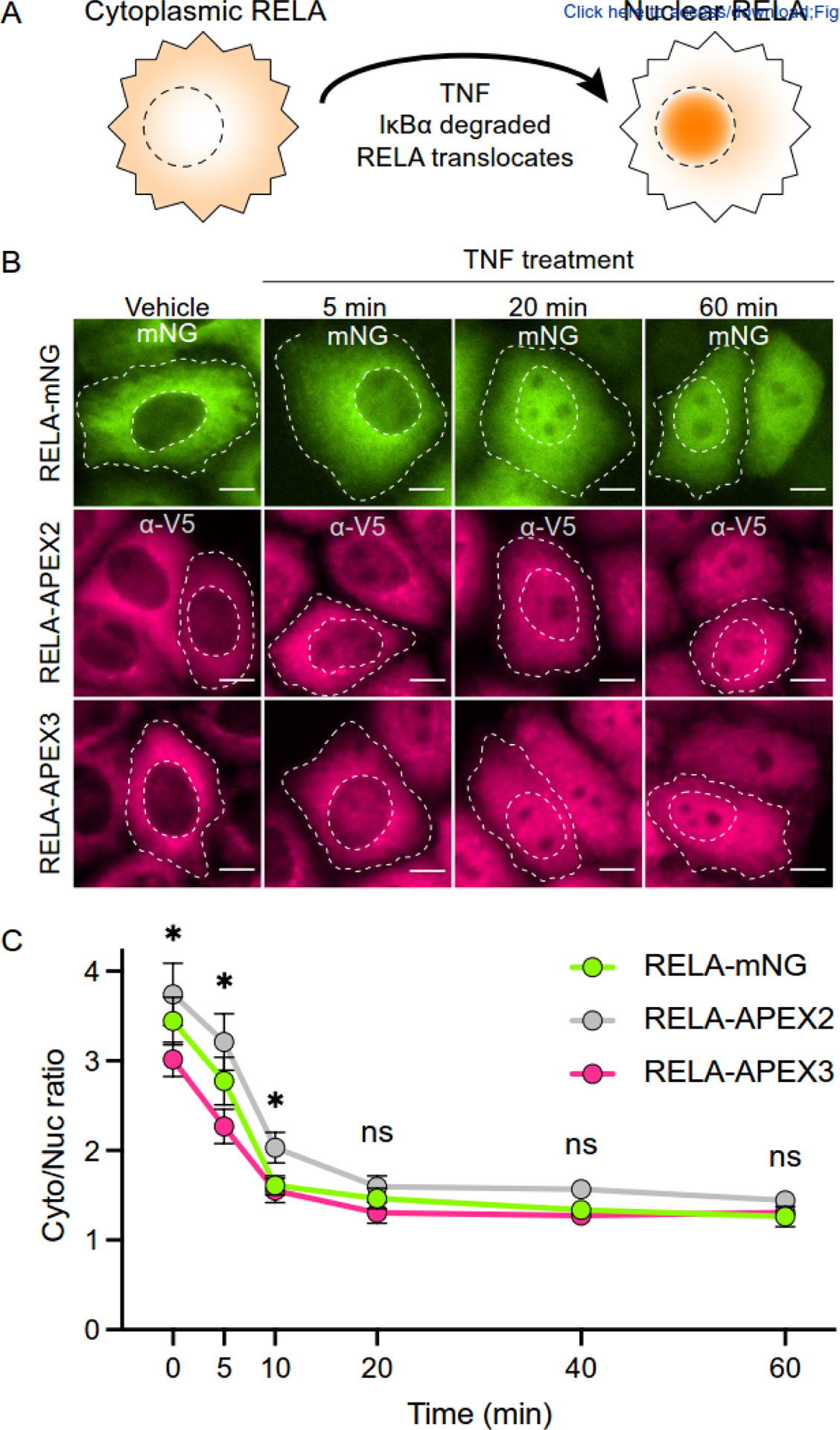
APEX3 localizes and functions appropriately as fusion to RELA. (**A**) Schematic of RELA relocalization following TNF stimulation. (**B**) Representative images of fixed HeLa cells stably expressing RELA-mNG, RELA-APEX2-V5, or RELA-APEX3-V5 following vehicle control or 30 ng/mL TNF treatment for the indicated times (green, mNG fluorescence; rose, anti-V5 immunostaining; scale = 10 μm). Cell and nuclear boundaries outlined with white dashed lines. (**C**) Quantification of cytoplasmic-to-nuclear fluorescence ratio of cells in panel B (N=50 cells per condition; ns, not significant; *, *p* < 0.001 by two-way ANOVA).

Finally, the functionality of APEX3 was tested by fusing it to RELA, which is a well-characterized nucleocytoplasmic shuttling protein (reviewed by [50,51]). RELA is a key component of the NF-κB transcription factor complex that exhibits cytoplasmic localization at steady state when bound by its inhibitor IκBα [52–54]. However, upon receiving a relevant stimulus (*e.g*., LPS, IL-1, or TNF), IκBα releases from RELA which unveils a NLS that allows translocation to the nucleus, DNA binding, and interaction with transcription elongation factors, CCNT1 and CDK9 (schematic in Figure 4A). Therefore, human RELA was fused to APEX2 or APEX3 with a V5 epitope to observe localization and labeling at steady state and following stimulation with TNF for multiple time points in addition to RELA-mNG as a control (Figure 4B; with quantification in Figure 4C). As expected, RELA-mNG, RELA-APEX, and RELA-APEX3 exhibit cytoplasmic localization at steady state. Moreover, all 3 constructs fully relocalize to the nucleus upon stimulation with TNF after 60 minutes (min). However, by performing a time-course of TNF treatment, we observed that RELA-mNG and RELA-APEX3 exhibit faster nuclear translocation relative to RELA-APEX2 (Figure 4B; quantified in Figure 4C; *p* < 0.001 by two-way ANOVA). Together these results support the use of APEX3 as an optimized tool for rapid *in cellulo* proximity labeling, particularly for nucleocytoplasmic shuttling proteins or proteins with uncharacterized localization and regulatory mechanisms.

## DISCUSSION

Proximity labeling is now a widely used experimental technique for identifying molecular interactions across multiple time scales, subcellular locales, and organismal systems [28]. Selecting the appropriate enzymatic labeling tool can dramatically affect the nature and specificity of interactions identified. In trial experiments with the engineered ascorbate peroxidase, APEX2, we observed a cytoplasmic-biased localization that was sensitive to the XPO1 inhibitor, LMB (Figure 1). We identified an amino acid motif at the carboxy-terminus of APEX2 that can act heterologously as an NES when fused to mCherry (Figure 2). Mutations at hydrophobic residues of the NES-like motif in the mCherry fusion as well as in the context of full-length APEX2 eliminated NES-like activity, however only L242A retained the peroxide-dependent labeling activity (Figure 3). We called the L242A mutant, APEX3 – a localization-optimized ascorbate peroxidase. Finally, we confirmed that APEX3 faithfully localizes as a fusion to RELA during NF-κB signaling following TNF stimulation (Figure 4). APEX3 is therefore anticipated to enable a wider range of proximity labeling applications.

Previous work used APEX2 largely in the context of fusions to very strong subcellular-targeting amino acid motifs or to proteins with strong subcellular localizations. Notably, APEX2 fused to potent subcellular localization signals was used to determine the subcellular distribution of cellular RNA molecules [34]. Others used APEX2 fused to eIF4A1 to identify RNA and protein interactions during translation initiation and within stress granules [33]. As the primary helicase acting during translation initiation, eIF4A1 has a well characterized and strong cytoplasmic localization. In this study, all the APEX2 fusion constructs also included strong subcellular localization signals in addition to proteins of interest: NES derived from HIV-1 Rev on eIF4A1, eIF4E, and a GFP control, SV40 NLS on CBX1, and an endoplasmic reticulum signal from CYP2C1. Another group used G-protein coupled receptors (GPCRs) fused to APEX2 to identify protein-protein interactions that occur during signaling cascades with high spatiotemporal resolution [32]. As GPCRs are membrane-associated proteins, the NES_APEX2_ was unlikely to have biased their results. Others used APEX2 fused to two RNA-binding proteins (MS2 coat protein or dCas13a) engineered to include strong nuclear localization signals to tether APEX2 to RNAs of interest (bound by MS2 or dCas13a) and label endogenous proteins bound to those RNAs of interest [55]. Finally, two groups used APEX2 fused to a catalytically inactive Cas9 to label proteins associated with specific genomic loci [56,57]. Altogether, the interactomes identified in these reports using APEX2 are unlikely to have been markedly affected by the cytoplasmic-biased localization of APEX2 due to the strong localization determinants that effectively “over-ride” the putative NES of APEX2.

The subcellular localization of RNA and proteins determines their functions, interactions, and regulation. Methods that can identify the complex networks of interacting RNA and proteins within cells are crucial in understanding development, disease, and degeneration as well as finding therapeutic strategies to extend and improve health-spans and lifespans. Here, we have identified and systematically confirmed a putative NES within the APEX2 proximity labeling peroxidase that may complicate its use for identifying interaction networks for several different types of proteins (*e.g*., nuclear proteins and nucleocytoplasmic shuttling proteins). While many plant APX1 proteins are named “ascorbate peroxidase, cytosolic” as determined by cell fractionation followed by activity assay or western blot [58–62], our studies here are the first to identify and characterize this conserved putative NES, which we speculate is perhaps necessary for preventing promiscuous activity in the plant cell nucleus. APEX2 has been used over other *in cellulo* biotin labeling methods such as BirA*/BioID [63] or TurboID [64] due to its rapid labeling activity. However, we found that the NES-like activity within APEX2 noticeably slows nuclear translocation on a time-scale of minutes, compared to APEX3. Importantly, we recommend that researchers validate the subcellular localization of proteins of interest with minimal tags relative to their APEX2 fusion proteins, as is common for fluorescent fusion proteins. We encourage those using APEX, APEX2, or split-APEX2 to test APEX3 in their systems to reduce potential unforeseen complications due to active nuclear export. In addition, APEX2 and APEX3 could be used in parallel to generate comparative interactomes of a particular protein of interest.

It is remarkable how mutationally sensitive the APEX2 enzyme is regarding amino acid changes in the conserved NES-containing alpha-helix region. In addition, it is curious that the hydrophobic NES_APEX2_ residues that may interact with the nuclear export factor XPO1 are structurally occluded, which suggests dynamic flexibility in the structure of APEX2 throughout the cell. This carboxy-terminal alpha-helix of APEX2 could hinge away from the globular domain, which would allow binding to XPO1 and nuclear export. We would further predict that APEX2 would become transiently inactive during export but regain ascorbate peroxidase activity upon NES helix reorganization in the cytosol. Furthermore, it is possible that (1) post-translational modifications or (2) heme occupancy may modulate the accessibility of this region, or (3) a co-factor may facilitate an indirect interaction between APEX2 and XPO1. However, such post-translational modification machinery and substrate preferences or unknown co-factor for indirect interactions would need to be conserved in plants as well as in humans that lack an APX1 homolog. Finally, we note that while we have not shown a direct interaction between APEX2 and XPO1 here, our data strongly support this region of APEX2 exhibiting NES-like activity and, importantly, led to the rational discovery of APEX3.

## MATERIALS AND METHODS

### Plasmids

APEX2 cDNA was ordered as a gBlock from IDT based on sequence available from GFP-APEX2-NIK3x [34] (Addgene #129274) with synonymous codon optimization to remove restriction enzyme recognition sites and cloned in-frame to the V5 epitope (GKPIPNPLLGLDST) [65]. All APEX2-encoding (and mNeonGreen control) plasmid DNA constructs were cloned using conventional restriction enzymes and T4 DNA ligation (New England Biosciences, #M0202L) cloning into a bespoke MIGR1-derived simple retroviral vector [66] encoding blasticidin resistance gene downstream of an IRES. mNeonGreen was ordered as a codon optimized gBlock from IDT based on the published amino acid sequence of mNeonGreen [67]. Codon-optimized cDNA encoding human RELA (NCBI GenBank accession: NP_068810.3) was ordered as a gBlock from IDT. mCherry expression constructs were cloned by PCR amplification with primers encoding amino acid motifs at the carboxy-terminus of mCherry. All amino acid substitutions were generated by site-directed mutagenesis using Phusion DNA Polymerase. Amino acid sequences of mNG-APEX3-V5 and mCherry-NES_APEX2_ are provided in Figure S4.

### Cell culture, transfections, and transductions

HeLa and 293T cells were obtained from ATCC and cultured in DMEM supplemented with 10% fetal bovine serum (FBS) and 1% penicillin/streptomycin at 37°C/5%CO_2_/50%H_2_O. All transfections were performed using TransIT-LT1 (Mirus Bio, #MIR-2306) with OptiMEM serum-free media at the following ratio: 100 μL OptiMEM, 3 μg LT1, and 1 μg DNA. To generate retrovirus for transducing APEX2 expressing vectors, pre-adhered 293T cells in 6-well plates were transfected with 1 μg APEX2 or control package plasmid, 1 μg pMD.Gag/GagPol [68] plasmid, and 200 ng VSV-G [69] plasmid. Media was replaced at 24 hrs post-transfection. Virus-containing supernatant was harvested at 48 hrs post-transfection, 0.45 μm syringe-filtered, and stored at −20°C. Stable cells were generated as described [70]. Briefly, approximately 2,500 target cells (HeLa or 293T) were seeded into a 96-well flat bottom plate, allowed to adhere overnight at 37°C/5%CO_2_/50%H_2_O, and 50–200 μL of transducing viral supernatant with 10 μg/mL polybrene added to each well. Transduced cells were selected at 48 hrs post-transduction with 2 μg/mL Blasticidin S (GoldBio, #B-800-100), expanded, and maintained in culture in the presence of drug. Leptomycin B (LMB; Sigma; #L2913, dissolved in methanol) was used at a final concentration of 12.5 nM for two hrs. RELA-APEX/mNG expressing cells were stimulated with TNF (R&D Systems, #210-TA-020; 30 ng/mL) for up to 60 minutes.

### Peroxide-dependent proximity labeling

For APEX2 labeling experiments, cells were plated in culture media for 24 hrs, then replaced with culture media containing 500 μM biotin-phenol (Iris Biotech GMBH; #LS3500) and incubated for at least 60 minutes. For peroxide-dependent labeling, H_2_O_2_ (Sigma; #H1009) was added to culture media at 100 mM concentration for 60 seconds while gently shaking/swirling. Peroxidase labeling reaction was stopped by removing media and replacing with “quenching solution” containing 10 mM sodium ascorbate (Sigma; #A7631), 10 mM sodium azide (Sigma; #S2002), and 5 mM Trolox (Sigma; #238813) in PBS. Cells were washed twice more with quenching solution. For fluorescence-based detection of labeling, cells were washed with PBS prior to fixation with 4% paraformaldehyde for 20 minutes and washed again with PBS.

### Immunostaining and fluorescence microscopy

Fixed cells were washed with PBS three times prior to permeabilization in PBS containing 0.25% Triton X-100 for 15 minutes. After washing three times with PBS, cells with blocked with PBS containing 3% bovine serum albumin (blocking buffer) for one hour. Cells were incubated with primary antibodies detecting V5 epitope (mouse anti-V5; Invitrogen, #R960-25) in blocking buffer for one hour at room temperature or overnight at 4°C at a 1:1000 dilution. Cells were washed thrice with PBS and incubated with secondary antibodies in blocking buffer for one hour at room temperatures at the following dilutions: anti-mouse AlexaFluor-568 and streptavidin AlexaFluor-647 each at 1:1000 (Invitrogen; #A11032 and #S32357). Cells were washed once with PBS, incubated with PBS containing DAPI (Sigma; #D9542; 0.1μg/mL) for 15 mins, then washed twice with PBS. We note that imaging fixed and permeabilized cells expressing mNeonGreen exhibited a different distribution than live cells, yet other cells did not exhibit remarkable differences in fixed versus live cells. Cells were imaged using a Nikon Ti-2E widefield microscope using a 20X objective (NA 0.75). Live cell imaging of mNeonGreen-tagged constructs was performed similarly on a Nikon Ti-2E widefield microscope. LMB treatment was performed on live cells for 2 hrs at 12.5 nM alongside vehicle treated cells and/or cells imaged prior to treatment. Images were processed and analyzed using FIJI [71]. Cytoplasmic-to-nuclear ratio was quantified using integrated fluorescence intensity from each compartment for at least 50 cells per condition. Whole cell streptavidin integrated fluorescence intensity was quantified for at least 50 cells per condition and normalized to the maximum value quantified for wild-type APEX2. All constructs were tested in biological triplicate. Representative images are displayed with mNeonGreen in green, V5 staining in rose, streptavidin staining in blue, and mCherry in magenta. All scale bars represent 10 μm.

### Bioinformatic analysis

Sequence logo of NES peptides from UniRef50 for UniProt P48534 containing 266 trimmed UniProt entries with ≥50% identity to *Pisum sativum* APX1 amino acid sequence (GenBank accession: AAA33645). Amino acid sequences were aligned using ClustalOmega [72] in SeaView5 [73]. Aligned sequences corresponding to residues 237 to 249 from *Glycine hispida* APX1 (GenBank accession: AAD20022) were used to generate the sequence logo using WebLogo [74] (version 2.8.2).

### Statistical analyses

GraphPad Prism 9.0 was used for statistical analysis (one-way or two-way ANOVA with corrections for multiple comparisons) of quantitative data presented as mean +/− 95% confidence interval for cytoplasmic-to-nuclear fluorescence intensity ratio or streptavidin intensity.

## Supplementary Material

1

2

3

4

5

## HIGHLIGHTS

- APEX2 peroxidase has cytoplasmic localization due to a putative NES
- Most mutations that eliminate NES activity also disrupt peroxidase function
- One separation-of-function mutant, L242A, shows unbiased cell-wide localization
- APEX3 (APEX2-L242A) is a more versatile proximity labeling enzyme

## ABBREVIATIONS:

APEX2/3: ascorbate peroxidases
IP: immunoprecipitation
LMB: leptomycin B
mNG: mNeonGreen
NES: nuclear export signal
NLS: nuclear localization signal
POI: protein of interest
PPI: protein-protein interaction
RBP: RNA binding protein
RPI: RNA-protein interaction
XPO1: Exportin-1
