# Supplementary material for: APEX3 – an optimized tool for rapid and unbiased proximity labeling
Source: J Mol Biol. Author manuscript; Available in PMC 2023 Jul 1. (PMC10247536; doi:10.1016/j.jmb.2023.168145)
Supplement: 5 [file NIHMS1900932-supplement-5.docx]

**Figure S4**

mNG-APEX3-V5 amino acid sequence

mNG

APEX3 (NES is underlined and L242A is bold)

V5

MVSKGEEDNMASLPATHELHIFGSINGVDFDMVGQGTGNPNDGYEELNLKSTKGDLQFSPWILVPHIGYGFHQYLPYPDGMSPFQAAMVDGSGYQVHRTMQFEDGASLTVNYRYTYEGSHIKGEAQVKGTGFPADGPVMTNSLTAADWCRSKKTYPNDKTIISTFKWSYTTGNGKRYRSTARTTYTFAKPMAANYLKNQPMYVFRKTELKHSKTELNFKEWQKAFTDVMGMDELYKGGGGSGGGGTRTGGGGGSGKSYPTVSADYQDAVEKAKKKLRGFIAEKRCAPLMLRLAFHSAGTFDKGTKTGGPFGTIKHPAELAHSANNGLDIAVRLLEPLKAEFPILSYADFYQLAGVVAVEVTGGPKVPFHPGREDKPEPPPEGRLPDPTKGSDHLRDVFGKAMGLTDQDIVALSGGHTIGAAHKERSGFEGPWTSNPLIFDNSYFTELLSGEKEGLLQLPSDKALLSDPVFRPLVDKYAADEDAFFADYAEAHQK**A**SELGFADAGGGGSGKPIPNPLLGLDST*

mCherry-NES_APEX2_ amino acid sequence

mCherry

NES_APEX2_ (NES is underlined)

MVSKGEEDNMAIIKEFMRFKVHMEGSVNGHEFEIEGEGEGRPYEGTQTAKLKVTKGGPLPFAWDILSPQFMYGSKAYVKHPADIPDYLKLSFPEGFKWERVMNFEDGGVVTVTQDSSLQDGEFIYKVKLRGTNFPSDGPVMQKKTMGWEASSERMYPEDGALKGEIKQRLKLKDGGHYDAEVKTTYKAKKPVQLPGAYNVNIKLDITSHNEDYTIVEQYERAEGRHSTGGMDELYKEAHQKLSELGFAD*
